# Beyond Thrombopoiesis: The Immune Functions of Megakaryocytes in Bacterial Infections and Sepsis

**DOI:** 10.3390/ijms262211191

**Published:** 2025-11-19

**Authors:** Marina Leardini-Tristão, Meenakshi Banerjee

**Affiliations:** 1 Molecular Medicine Program, University of Utah Health, Salt Lake City, UT 84112, USA; u6029302@utah.edu; 2 Division of Hematology and Hematologic Malignancies, Department of Internal Medicine, University of Utah Health, Salt Lake City, UT 84112, USA

**Keywords:** megakaryocytes, immune cells, bacteria, sepsis, platelet production, immune response, bacteria-host interaction

## Abstract

Megakaryocytes (MKs) are specialized hematopoietic cells long recognized for their ability to produce platelets. Increasing evidence now highlights MKs as multifunctional immune effectors that bridge hematopoiesis with host immunity. In the bone marrow (BM), MKs arise through thrombopoietin (TPO)-mediated differentiation of hematopoietic stem cells (HSCs) and show substantial heterogeneity, with discrete subsets specialized for platelet production (thrombopoiesis), HSC niche maintenance, or immune modulation. Outside the BM, MKs in the lungs and spleen perform tissue-specific immune functions, including pathogen recognition, phagocytosis, antigen presentation, and secretion of cytokines. During bacterial infections and sepsis, infectious or inflammatory cues reprogram MKs to amplify immune signaling and host responses, but can also drive coagulopathy and contribute to organ failure. Collectively, these findings redefine MKs as dynamic immunomodulatory cells positioned at the interface of thrombopoiesis and innate and adaptive immunity. In this review, we synthesize emerging literature on MK biogenesis, functional diversity, and immune modulation, with a special focus on their roles in bacterial infections and sepsis.

## 1. Introduction

Megakaryocytes (MKs) are essential, highly specialized precursor cells that produce platelets, which are critical mediators of hemostasis and thrombosis [1,2]. This platelet production process is known as thrombopoiesis, and it is defined as the maturation and differentiation of MKs from hematopoietic stem cells (HSCs), typically from the bone marrow (BM), resulting in the release of platelets in the bloodstream [3]. In the adult BM, MKs range from 20 to 100 μm in diameter, making them the largest and rarest cells, comprising up to 0.5% of the total BM cellularity, and are homogeneously distributed within the entire BM [4,5,6]. Mature MKs contain a single polyploid multilobulated nucleus with their DNA content ranging from 2 to 128 N, as a consequence of a unique phenomenon called endomitosis, during which the nucleus increases its DNA content without cytokinesis [7,8,9]. The cytoplasmic mass of the MK also increases proportionally to its ploidy status following this process of polyploidization, which is necessary for efficient platelet production [7].

The cytoplasm of a mature MK is enriched with secretory granules (alpha granules, dense granules, and lysosomes), mitochondria, endosomes, endoplasmic reticulum, and the Golgi apparatus. A unique and defining ultrastructural feature of megakaryocytes is a vast labyrinth of membrane invaginations derived from its plasma membrane called the demarcation membrane system (DMS). The DMS serves as a source of plasma membrane for nascent platelets, providing the surface area required to form thousands of platelets per megakaryocyte [10,11]. Newly formed platelets are released from MKs via cytoplasmic protrusions called proplatelets that project into the lumen of BM sinusoids, where they fragment and shed platelets into the circulation [12].

Beyond their classical role in platelet production, or “thrombopoiesis,” accumulating evidence underscores the multifaceted functions of MKs within the BM microenvironment. MKs have been shown to contribute to (1) the maintenance of the BM niche by supporting HSC functions, and (2) the regulation of innate immunity and inflammation by functioning as “immune cells” [13,14]. Furthermore, MKs have been used as surrogate models for platelets in experimental settings, owing to their suitability for gene-editing approaches, such as clustered regularly interspaced short palindromic repeats (CRISPR)-Cas9 [15,16]. Unlike anucleate platelets, which cannot be directly genetically modified, MKs provide a practical alternative, as they exhibit platelet-like phenotypic responses, e.g., agonist-induced granule cargo secretion [15].

Given these diverse roles of MKs, it is particularly relevant to investigate how MK biology is altered during inflammatory settings such as sepsis. Sepsis is defined as organ dysfunction resulting from a dysregulated immune response to systemic infection and remains a leading cause of mortality worldwide [17]. In 2020, a global analysis reported 48.9 million sepsis cases and 11 million related deaths, representing approximately 20% of all global deaths [18]. Of all the forms of sepsis, bacterial infection-induced sepsis is the most common and results in inflammation, endothelial dysfunction, sepsis-induced coagulopathy (SIC), and ultimately, multiorgan failure and death [19].

Although the role of platelets has been well-established in sepsis, growing evidence suggests that MKs may also contribute to the physiology and pathophysiology of sepsis [20]. In this review, we will discuss the landscape of MK biogenesis, highlight their expanding functional diversity, and emerging roles during bacterial infections and in clinical inflammatory syndromes such as sepsis.

## 2. Megakaryocyte Biogenesis and Heterogeneity

Within the adult BM, HSCs commit to MK biogenesis by sequentially differentiating from multipotent progenitors (MPPs) along the myeloid lineage via common myeloid progenitors (CMPs), then through megakaryocyte-erythroid progenitors (MEPs) [21,22]. MEPs subsequently give rise to megakaryocyte precursors (MkPs), which mature into functional MKs. This tightly regulated, multistep process is primarily governed by the liver-derived hormone, thrombopoietin (TPO) [23]. TPO engages its cognate receptor, the myeloproliferative leukemia protein (c-Mpl), which is expressed on HSCs, multipotent progenitors, MEPs, MKPs, mature MKs, and platelets, to drive MK differentiation and platelet production [24,25,26,27]. TPO levels, in turn, are regulated by the (1) concentration of circulating platelets through negative feedback, (2) binding of senescent platelets to the hepatic asialoglycoprotein Ashwell–Morell Receptor (AMR), and (3) by the GPIbα receptor on platelet surfaces [28,29,30].

While the TPO-c-Mpl axis remains the predominant route of MK biogenesis, MK production still occurs even in its absence. Other inflammatory cytokines can also induce MK expansion. For instance, systemic inflammation generates the pro-inflammatory cytokine, interleukin-6 (IL-6), which can trigger acute-phase MK production and increased platelet production, also called thrombocytosis [31]. Regulated on activation, normal T cell expressed, and secreted (RANTES) is a cytokine stored and released by platelets, among other cells, upon activation during infectious or inflammatory conditions, which can induce MK biogenesis and proplatelet production [32]. Other cytokines, such as stromal cell-derived factor-1 (SDF-1), which is secreted by endothelial cells, immature osteoblasts, and mesenchymal stromal cells, and fibroblast growth factor-4 (FGF-4), also secreted by mesenchymal stromal cells, can facilitate the production of MKs in the BM [33].

**Figure 1:**
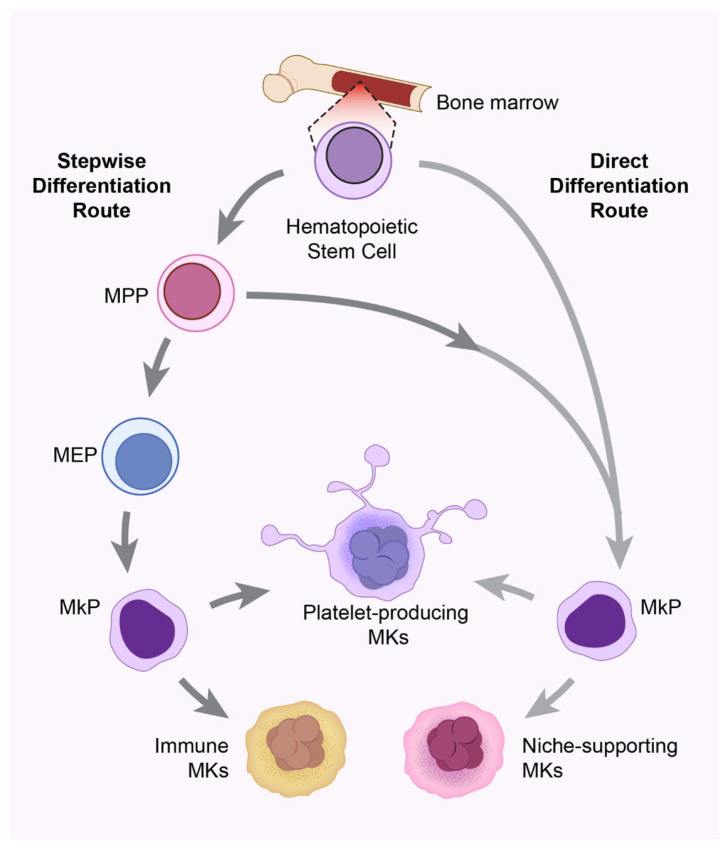
Route of differentiation dictates megakaryocyte function in the bone marrow. In the bone marrow (BM), the generation and function of megakaryocytes depend on their route of differentiation from primitive hematopoietic stem cells (HSCs). MKs, which are generated via the stepwise route or the sequential differentiation of HSCs-Multipotent Progenitors (MPPs)-Megakaryocytic-Erythroid Progenitors (MEPs)-Megakaryocyte Progenitors (MkPs) to finally mature MKs, are thought to participate in immune functions (i.e., “immune MKs”) in addition to being “platelet-producing” MKs. The other route, called the direct route, involves HSCs (or MPPs) differentiating directly in one step to MkPs, thereby generating mature MKs without passing through the intermediate steps of differentiation. These direct route MKs support the BM niche for HSC homeostasis and are called “niche-supporting MKs” in addition to being “platelet-producing” MKs.

Advances in single-cell omics have revealed substantial heterogeneity among MKs, uncovering distinct subsets with specialized functions. Intriguingly, these studies have proposed the novel concept that the route of MK differentiation from HSCs dictates their functional phenotype [22,34]. MKs, which are generated via the “stepwise route” or the sequential differentiation of HSCs-MPPs-MEPs-MkPs-MKs, as explained earlier, are thought to participate in immune functions (i.e., “immune MKs”) in addition to being “platelet-producing” MKs (**Figure 1**) [35,36]. This stepwise route of differentiation is triggered upon platelet consumption or inflammatory cues [36]. The other route, called the “direct route”, involves HSCs (or MPPs) differentiating directly in one step to MkPs, thereby generating mature MKs without passing through the intermediate steps of differentiation [37,38,39]. These MKs, generated via the direct route, are thought to support the BM niche for HSC homeostasis and are called “niche-supporting MKs” (**Figure 1**). The direct route operates under myeloablative stress and thrombocytopenic conditions [39]. These HSC niche-supporting MKs can also produce platelets. Li et al. identified that CD48^high^ HSCs/MkPs generate MKs via the stepwise route, whereas CD48^low^ HSCs/MkPs generate MKs via the direct route to generate functionally distinct MKs [40]. In addition to these routes of MK biogenesis, a subset of HSCs displays lineage bias towards megakaryocytes [41]. These MK-biased HSCs are typically found within the CD150^high^-CD41^+^-vWF^+^ HSC compartment. They are transcriptionally primed for MK differentiation as evidenced by the expression of early MK genes such as *Itga2b (CD41)*, *Vwf*, *Pf4*, and *Fli1* [41].

Moreover, Sun and colleagues used single-cell transcriptomics on murine BM MKs and freshly isolated primary human BM MKs to demonstrate four distinct subsets of MKs, each with a unique function, characterized by gene signatures related to platelet generation, HSC niche maintenance, active cycling, and immune functions [42]. The authors uncovered: (1) **Immune MKs** (low ploidy, 2N–8N) were enriched for immune cell markers such as lymphocyte-specific protein 1 (LSP1) and leukocyte surface antigen CD53 and displayed phagocytic and antigen-presenting capacity. These cells upregulated transcription factors associated with myeloid differentiation, including *Spi1*, *Cebpd*, *Irf5*, and *Irf8*, but lacked the potential to produce platelets. (2) **HSC niche-supporting MKs** (intermediate ploidy, 8N–32N) expressed *Igf1*, *Pf4*, and Wnt pathway genes (*Wnt3a*, *Wnt4*, *Dkk1*, *Dkk2*) involved in HSC regulation. (3) **Platelet-producing MKs** (high ploidy, >32N) showed strong expression of genes required for thrombopoiesis and hemostasis, including *Tubb1*, *Myh9*, *Vwf*, *Gp1ba*, *Gp5*, *Gp6*, *P2ry1*, and *P2ry12*. (4) **Active cycling/polyploidizing MKs** (2N–32N) were enriched for DNA replication genes (*Pola2*, *Pold2*) and cell-cycle regulators (**Table 1**).

Liu et al., in their 2021 paper, echoed similar findings, wherein they identified cellular heterogeneity between human MK subpopulations using single-cell transcriptomics profiling [43]. In their studies, they identified two surface molecules, CD148 and CD48, as markers of “immune-responsive” MKs. These CD148^+^CD48^+^ MKs respond rapidly to immunogenic stimuli both in vitro and in vivo, expressing immune receptors and mediators such as inflammation-associated genes *S100a11* and *S100a12*, *Tnf*, *Tnfaip3*, *Tlr2*, and *Tlr4*. In a mouse model of *Escherichia coli* infection, the authors noted that the proportion of CD148^+^ CD48^+^ MKs increased post-infection. These MKs expressed high levels of several pattern-recognition receptors, including *CD88*, *Fpr1* (Formyl peptide receptor 1), and toll-like receptor (TLR)-2 and 4. Furthermore, these CD48^+^ MKs were found localized closer to blood vessels in the mouse BM after immune challenges compared to the CD48^-^ MKs, suggesting a potential role in mediating “immune surveillance” during acute infection.

Collectively, these findings underscore the remarkable heterogeneity of MKs within the adult BM. Infectious and inflammatory states can further contribute to this heterogeneity [44,45]. These distinct subsets of MKs harbor specialized transcriptional programs and functional attributes, enabling them to perform diverse roles in platelet biogenesis, host immune response, and HSC niche regulation.

## 3. Tissue-Specific Features of Megakaryocytes

Beyond the BM, megakaryocytes have been found in the lungs, spleen, and peripheral blood in adult mice and humans [46,47,48,49,50]. Location- and tissue-specific effects introduce another layer of complexity to MK functions.

### 3.1. Lung Megakaryocytes

MKs within the lung vasculature were first described by Aschoff in 1893 [51]. Subsequent studies have confirmed that mature MKs circulate and lodge within pulmonary vascular beds, serving as a local source of platelet production during infection and inflammation [46,52]. Pulmonary MKs act as immune sentinels by trapping pathogens within the microvasculature, releasing platelets enriched with antimicrobial peptides, and rapidly secreting inflammatory mediators in response to microbial cues [53].

Morphologically, lung MKs are smaller than their BM counterparts, and pathophysiological states (e.g., infections, sepsis) determine their numbers [54]. Dual-labeling analyses of CD41^+^ MKs isolated from lungs reveal that MKs reside in both intravascular and extravascular compartments [46,52]. The majority localize extravascularly within the pulmonary interstitium, and a smaller subset of MKs is found intravascularly and thought to participate in platelet generation, especially under infectious/inflammatory conditions [47,52]. Lung MK ploidy remains a debated topic: some studies report predominantly low-ploidy (2N) cells, while others detect rare high-ploidy (>16N) MKs coexisting with low-ploidy populations [52,55].

**Figure 2:**
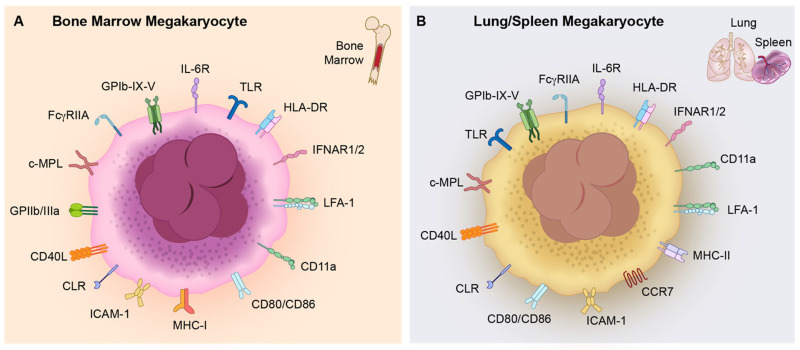
Repertoire of immune receptors on bone marrow and extramedullary megakaryocytes. (**A**). Bone marrow (BM) megakaryocytes (MKs) have surface receptors that support thrombopoiesis and immune sensing within the hematopoietic niche. These include the thrombopoietin receptor, c-Mpl, platelet glycoprotein complexes glycoprotein (GP) GPIb-IX-V and GPIIb/IIIa, and adhesion molecules ICAM-1 and LFA-1 and CD11a. BM MKs also express several immune receptors- FcγRIIA, TLRs, HLA-DR, IFNAR1/2, IL-6R, CD40L, CLR, CD80/CD86, and MHC Class I- highlighting their potential for pathogen recognition and response as well as cytokine release. (**B**). Extramedullary MKs, found in lungs and spleen, by comparison, show an enrichment in the expression of immune and migration markers, consistent with their proposed predominantly “immunomodulatory” roles. While these MKs retain key platelet and immune markers, common to BM MKs (GPIb-IX-V, GPIIb/IIIa, ICAM-1, LFA-1, CD11a, FcγRIIA, TLRs, HLA-DR, IFNAR1/2, IL-6R, CD40L, CLR, CD80/CD86), they also uniquely express CCR7 and MHC Class II, suggesting enhanced antigen-presentation and trafficking capabilities. Together, these profiles highlight the distinct tissue-specific functional specializations of MKs.

Despite tissue-specific differences in the number and size of lung MKs compared to BM MKs, several groups have utilized transcriptomic profiling to gain a better understanding of the functional landscape of these two types of MKs. Lung MKs are enriched in immune and inflammatory gene programs, including TLRs and chemokine receptors, whereas BM MKs preferentially express receptors involved in platelet biogenesis and hemostasis (**Figure 2**, **Table 2**) [46,52,54,55]. Given that the lungs represent a frontline barrier to airborne and blood-borne pathogens, these transcriptional features position lung-resident MKs as specialized immune effector cells.

Functional evidence supports this concept. Notably, using a murine model of bacterial pneumonia, Lefrancais et al. in 2017 found that lung MKs are functionally adapted for “immune surveillance” and can respond dynamically to infections and commit to pathogen clearance [46]. Pariser et al. in 2021, similarly showed that murine lung MKs, but not BM MKs, had gene expression patterns like those of professional antigen-presenting cells (APCs) [52]. Lung MKs, which they termed MK_L_, expressed major histocompatibility complex (MHC) class II (antigen-presenting markers) and CD11c (a dendritic cell marker) at higher levels. MHC II on lung MKs enables cross-presentation of ovalbumin peptides to CD4^+^ T cells, thereby promoting T cell activation and proliferation [52]. Certain immune receptors, such as CCR7, are only present on lung MKs, further underscoring the enhanced “immune” potential of lung MKs over BM MKs (**Figure 2**, **Table 2**).

### 3.2. Spleen Megakaryocytes

The spleen is an important site of extramedullary hematopoiesis [48]. Not surprisingly, MKs are found within the splenic vascular beds. HSCs have been shown to migrate out of their BM niches to the spleen in response to specific physiological or pathological cues (e.g., inflammation, thrombocytopenia, myelofibrosis), thereby generating splenic MKs [72,73]. These splenic MKs are found primarily within the red pulp and marginal zones of the spleen, often near the splenic sinusoids to facilitate platelet release [74]. Under steady-state conditions, splenic MKs are rare. However, their frequency increases during inflammation or infection-induced extramedullary hematopoiesis [73]. This contributes to extramedullary thrombopoiesis, compensating for the loss of platelet numbers common during stress- or sepsis-induced thrombocytopenia [75].

Functionally, splenic MKs more closely resemble lung MKs than BM MKs. Using single-cell RNA sequencing, Valet et al. found that splenic MKs possess distinct “immunomodulatory” phenotypes compared to BM MKs [75]. Splenic MKs express both MHC class I and II, costimulatory molecules such as CD40L, CD80, and CD86, and a range of pattern-recognition receptors, including TLRs (**Table 2**). The splenic MKs can phagocytose bacterial pathogens and process antigens to present them to CD4^+^ T cells, thereby functioning as antigen-presenting cells (APCs). Notably, increased expression of CD40L on platelets generated from “immune-skewed” splenic MKs has been shown to induce NETosis from neutrophils under settings of bacterial infections and sepsis [76].

In addition to the presence of surface immune receptors, splenic MKs release cytokines such as IL-6, IL-1β, tumor necrosis factor alpha (TNF-α), IFN-β, and platelet factor-4 (CXCL4/PF4) in response to inflammatory stimuli [77]. These mediators modulate both innate immune responses and HSC activity under infectious or inflammatory stress. During bacterial lipopolysaccharide (LPS) challenge or sepsis, splenic MKs upregulate inflammatory gene transcriptional programs, exacerbating systemic inflammation and contributing to sepsis-induced coagulopathy [75,78,79].

### 3.3. Circulating Megakaryocytes in Peripheral Blood

Since advances in imaging cytometry, single-cell RNA sequencing, and microfluidics, finding megakaryocytes in circulation has become increasingly feasible and no longer considered an artifact [49]. Although rare in frequency (only about 1–5 per mL of whole blood) [80], circulating MKs originate from mature BM MKs or MkPs, which transmigrate through the sinusoidal endothelium into circulation. This MK egress and homing is governed by the CXCR4-CXCL12, integrin (β1/β3), and Sphingosine-1-phosphate (S1P) signaling axes [33,81,82,83]. During sepsis, circulating MK counts are elevated [20]. Functionally, circulating MKs are thought to have egressed from the BM and are *en route* to distal organs, such as the lungs and spleen, where they participate in platelet production during infection [20,75]. While in circulation, they serve as immune sentinels, allowing MKs to sense pathogens and inflammatory cues, as they express several pattern-recognition receptors (PRRs) such as TLRs, MHC class I/II, and produce immunomodulatory cytokines that allow them to interact with neutrophils, monocytes, facilitating NETosis, leukocyte recruitment, and microvascular thrombosis [77,84].

## 4. Immune Functions of Megakaryocytes

Megakaryocytes express several immune receptors that sense pathogenic and inflammatory cues, enabling them to respond by secreting cytokines, chemokines, and other immune mediators (Figure 2, Table 2).

### 4.1. Megakaryocyte Immune Receptors

Several pattern-recognition receptors, including TLRs 1, 2, 4, 6, 7, and 8, are present on the surface of MKs in humans, which enable them to recognize pattern-associated molecules found on pathogenic bacteria and viruses [85]. Lung MKs typically have elevated expression of TLRs compared to BM MKs due to their potent immunogenic roles [52]. So far, studies have shown that TLR2 and TLR4 activation accelerate MK maturation and proplatelet formation, whereas TLR7 activation impairs megakaryocyte and platelet development [86]. Roles of the other TLRs remain under investigation.

Like platelets, human MKs express the low-affinity IgG receptor, FcγRIIA, which recognizes immune complexes [69]. Subsets of MKs express the high-affinity FcγRI receptor and are thought to stimulate MK-derived microparticle release [87]. Among the other canonical immune receptors expressed on MKs, CD40L expression enables MKs to communicate with B cells and macrophages [63]. Mature BM MKs also express MHC class I machinery to internalize and process antigens for cross-presentation to T cells, whereas lung MKs but not mature BM MKs express MHC class II molecules [54,59]. These MHC class I-antigen complexes can be transferred to platelets during proplatelet formation, allowing them limited antigen-presentation capabilities [52,88,89]. Interestingly, platelets express only MHC class I, not class II, further underscoring the predominant role of BM MKs in generating the bulk of circulating platelets [88,89].

### 4.2. Megakaryocyte Immune Mediators and Responses

**Figure 3:**
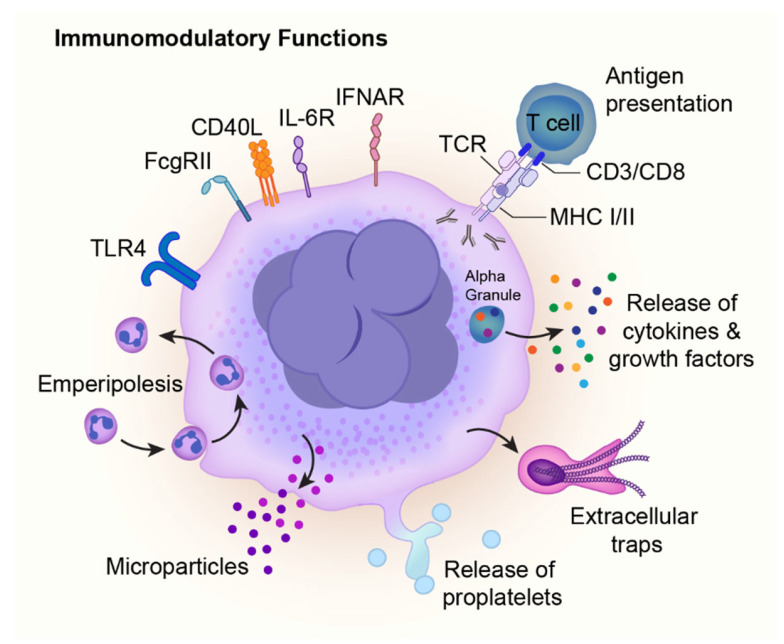
Immunomodulatory functions of megakaryocytes. Megakaryocytes (MKs) play diverse roles in the immune system beyond thrombopoiesis. They express immune receptors such as TLR, FcγRIIA, IL-6R, and IFNAR1/2, enabling responses to pathogenic and inflammatory triggers. Through MHC class I and II molecules, MKs can present antigens to T cells via TCR-CD3/CD8 interactions, thereby modulating the adaptive immune response. MKs also regulate their microenvironment through emperipolesis, which is the engulfment of leukocytes. MKs release cytokines and growth factors from their alpha granules and microparticles, in addition to their canonical function of releasing platelets. Moreover, in settings of bacterial infection and sepsis, MKs can also release their chromatin material as extracellular traps. Collectively, these mechanisms highlight MKs as versatile immune effector cells capable of orchestrating both innate and adaptive immune responses.

Megakaryocytes harbor numerous cytokines, chemokines, and other immune mediators, which are stored within their granules (**Figure 3**). MKs release their granule content into the BM microenvironment to mediate HSC functions and interact with other immune cells. Cytokines such as CXCL4/PF4 and transforming growth factor beta (TGFβ) regulate HSC proliferation and restrict MK maturation [90,91,92]. They also affect neutrophils, macrophages, and endothelial cells, which express CXCR3B and TGFβR [93,94,95]. Notably, CXCL4/PF4 and TGFβ activate neutrophils to promote migration [96,97,98]. MKs also release chemokines such as CXCL1 (KC) and CXCL2/macrophage inflammatory protein (MIP-2) to facilitate the migration of neutrophils out of the BM microenvironment [99].

Human MKs, like their murine counterparts, release cytokines such as PF4/CXCL4, TGFβ, CXCL1, IL-8, IL-1α/β, IL-6, and a proliferation-inducing ligand (APRIL) to regulate megakaryopoiesis and platelet production in addition to immune-mediated functions [46,87,100,101,102,103,104]. Moreover, human megakaryocytes can also release IL-1α/β, either directly or via microparticles (**Figure 3**) [102]. MK-derived microparticles express CD41, CD42b, Phosphatidylserine (PS), but lack CD62P and lysosome-associated membrane protein 1 (LAMP-1) [105,106]. MK microparticles also express both GPVI and C-type lectin-like receptor 2 (CLEC-2) and are thought to be the most prevalent type of microparticles in circulation [107]. A central distinguishing feature between platelet-derived microparticles and MK-derived microparticles is the presence of full-length filamin A in the MK-derived ones versus the cleaved filamin A present in platelet-derived microparticles [106].

In an intriguing phenomenon, MKs can internalize neutrophils and other leukocytes via a process called emperipolesis (Figure 3) [108,109,110,111]. Although very few MKs exhibit emperipolesis, this phenomenon is increased in pathologic conditions such as hematologic malignancies and myelofibrosis [109,112,113,114]. In experimental settings of LPS and IL-6 challenge, emperipolesis has been shown to increase in murine MKs [104,115]. Although the exact role of emperipolesis remains unknown, it may serve as a mode of cellular communication between MKs and leukocytes before egress from the BM.

Using in vitro techniques, studies have also demonstrated that MKs and Meg-01 cells exhibit chemotaxis, interact with bacteria, and release their intranuclear chromatin material in response to live *E. coli* [20]. The released nuclear material in the form of chromatin nets is very similar to neutrophil-derived extracellular traps. It contains histones and DNA and traps bacteria, potentially facilitating the clearance of pathogens [20].

## 5. Megakaryocytes in Bacterial Infections and Sepsis

Given the repertoire of immune functions MKs can perform, they have been implicated in various bacterial infections (summarized in **Table 3**). Some bacteria are primarily thrombocytopenia-inducing, whereas others are thrombosis-inducing, depending on how they alter MK and platelet functions. In pneumonia caused by *hypermucoviscous Klebsiella pneumoniae*, bacterial-induced platelet activation leads to increased apoptosis and inhibited megakaryocyte maturation, resulting in significant thrombocytopenia and an increased risk of bleeding and mortality in these patients [116]. In infections caused by *Fusobacterium nucleatum*, excessive thrombosis is associated with aberrant MK maturation and differentiation, driven by PI3K/Akt signaling [117].

In *Pseudomonas aeruginosa* infections, MKs exhibit increased cell cytotoxicity, leading to increased thrombocytopenia via the p38 pathway [118]. In infections caused by *Bacillus anthracis*, thrombocytopenia is caused by downregulation of megakaryocytic differentiation marker *DACH1*, which inhibits polyploidization and thereby reduces platelet production [119]. Furthermore, MKs can internalize pathogenic bacteria (e.g., *E. coli.*) and process them for antigen presentation via their MHC class I and II receptors to activate T cells [20,52,54].

During systemic inflammation and sepsis, megakaryopoiesis undergoes profound remodeling. Proinflammatory cytokines (e.g., IL-6) and activation of TLR signaling by pathogen- and damage-associated molecular patterns (PAMPs and DAMPs) induce an emergency hematopoietic program characterized by increased proliferation of MK progenitors [31,45,120,121]. However, the resulting MKs often exhibit immature or dysplastic morphology and altered ploidy (skewed toward lower DNA content), indicative of stress hematopoiesis [45]. The resulting platelets generated from these “stressed” MKs are morphologically enlarged, more granular, and functionally hyperreactive [122,123]. The appearance of large granular platelets in the circulation thus serves as a peripheral marker of MK activation and dysregulated thrombopoiesis under inflammatory conditions.

Given that MKs are not a uniform population but rather a spectrum of functionally specialized subsets with disparate immune, thrombopoietic, and niche-supporting transcriptional programs, it is expectedly that distinct MK subsets show differential responses during infection and inflammation. The “thrombopoietic MKs” exhibit reduced maturation and altered ploidy, resulting in large granular, more immature platelets. The “immune MKs” expand under inflammatory stimuli to generate more “immune MKs” capable of enhanced cytokine secretion and antigen presentation. At the same time, the “niche-supporting MKs” also serve as a proliferative pool that expands in response to inflammatory stimuli [20,40,43,124].

During sepsis, hematopoietic stem and progenitor cells (HSPCs) egress from the BM to distal organs, particularly the spleen and lungs, to generate MKs locally [75]. Splenic MKs increase dramatically and act as immunomodulatory antigen-presenting cells, while the lung MKs proliferate and contribute to both platelet production and immune surveillance in the lung microvasculature [46,52,75]. This redistribution allows MKs to participate directly in immune defense beyond thrombopoiesis.

Middleton et al. and others have demonstrated how sepsis alters the transcriptional programs of both human and murine MKs [16,44]. Like platelets, MKs are responsive to infections and inflammation. Interferon-induced transmembrane protein 3 (IFITM3) expression, which is upregulated by systemic interferons, is increased in platelets from bacterial sepsis patients and is associated with increased platelet reactivity [16]. Septic MKs exhibit transcriptional reprogramming toward immune and inflammatory pathways, with upregulation of interferon response genes and reduced platelet maturation signatures, both of which impact platelet functional responses [16,44,79,125,126]. This is supported by recent single-cell RNA sequencing studies, which demonstrate that MK-derived immune programs persist in circulating platelet progeny and shape their functional responses in sepsis [127]. Platelets generated from sepsis-reprogrammed MKs exhibit increased “immune” phenotypes, release CD40L and high mobility group box 1 (HMGB1), which promote neutrophil extracellular traps (NET) formation, further contributing to immunothrombosis and microvascular injury [63,128,129].

While MK activation supports host defense, dysregulated MK responses may exacerbate sepsis pathogenesis [79]. In fact, sepsis is often associated with a hypercoagulable state and consumptive thrombocytopenia [130]. This reduction in platelet counts can, in turn, drive compensatory thrombopoiesis through inflammatory cytokine signaling [79]. Excessive cytokine production by MKs can amplify systemic inflammation, activate coagulation pathways, and lead to disseminated intravascular coagulation (DIC), resulting in microthrombosis and multi-organ failure [79,131,132]. Aberrant MK maturation and platelet dysfunction during sepsis impairs hemostasis and impedes the resolution of inflammation [126]. Patients with sepsis often exhibit thrombocytopenia alongside increased immature MKs in circulation and tissues, common hallmarks of maladaptive stress megakaryopoiesis [45,133,134].

## 6. Therapeutic Implications

From a therapeutic perspective, MKs and platelets are increasingly being explored as carriers of therapeutic and diagnostic cargos, such as cytokines, RNAs, and microparticles, for targeting HSPCs or modulating immune responses. Megakaryocyte-derived microparticles are the most abundant microparticles in the bloodstream and have been shown to deliver synthetic small RNAs (miRNAs and siRNAs) to HSPCs in vitro [135]. Functionally, MK-derived microparticles serve as an intercellular conduit between MKs and HSCs, influencing gene expression and lineage commitment in HSCs [135,136]. Also, intravenous administration of human MK microparticles transplanted into healthy or thrombocytopenic mice induced *de novo* platelet biogenesis, demonstrating target specificity and functional efficacy in vivo [137]. These approaches hold promise for therapeutic delivery, for example, in bleeding disorders and settings of systemic inflammation such as sepsis, where platelet numbers are compromised.

Several strategies to engineer genetically edited platelets from MKs or MK progenitors have been developed. Some of these methods include transfecting MK progenitors or CD34^+^ HSCs with viral vectors containing desired transgenes or using CRISPR-Cas9-mediated genetic deletion of specific genes [15,138,139]. Additionally, non-viral strategies have also been developed, such as lipid nanoparticles (LNPs), which contain messenger RNA (mRNA) of choice and achieve ~99% transfection efficiencies, without affecting MK maturation or viability [140].

In terms of therapeutics that have shown promising results in amplifying platelet production by directly affecting MKs or MK progenitors, Eltrombopag, a thrombopoietin mimetic, stimulates platelet production and is well-documented. In clinical trials, Eltrombopag was shown to improve platelet counts in patients with relapsed or refractory idiopathic thrombocytopenic purpura (ITP) (NCT00102739) [141]. Recently, Eltrombopag has been shown to be beneficial in improving platelet counts in critically ill patients with sepsis [142]. Ongoing clinical trials involving injecting cord blood-derived MKs to improve platelet counts in patients presenting with thrombocytopenia will shed more insight into the efficacy of MK-specific therapeutic strategies (NCT06534255, NCT02241031).

Despite their clinical relevance, isolation and culture of MKs remain challenging due to their fragility, large size, rarity, and storage issues [143]. The in vitro generation of MKs from human umbilical cord blood-derived CD34^+^ HSCs or inducible pluripotent stem cells (iPSCs) requires prolonged culturing under complex cytokine conditions, often resulting in variable donor-dependent differentiation capacity and overall yields of mature MKs [139,144]. Further investigation on improving MK yields will enable improved use for clinical and therapeutic settings.

## 7. Conclusions and Future Perspectives

Although classically recognized for their essential role in platelet biogenesis, MKs are now understood to possess diverse and multifaceted functions extending well beyond thrombopoiesis. Emerging evidence reveals that MKs actively modulate immune responses, support HSC niche homeostasis, and sustain a dynamic pool of cycling MKs within the BM microenvironment. Advances in bulk and single-cell transcriptional profiling have identified distinct MK subsets endowed with specialized transcriptional circuitry. MKs express immune receptors, release immune mediators and microparticles, and crosstalk with canonical immune cells. Further studies will clarify the tissue-specific effects of MKs, particularly their capacity to generate platelets and uncover novel immunomodulatory roles. Collectively, these features position MKs as bona fide immune cells that contribute to host defense, particularly during infections and sepsis.

In summary, MKs have emerged as highly versatile cellular effectors that bridge hematopoiesis, immunity, and thrombopoiesis. Continued investigation into their context-dependent functions and therapeutic applications will help redefine our understanding of these remarkable cells, both in health and disease.

## Figures and Tables

**Table 1:** Single-cell RNA Sequencing-derived Classification of Bone Marrow MK subsets (from Sun et al. 2021) [42].

MK Subset	Ploidy	Associated Markers	Functions
Immune MKs	2N–8N	CD53^+^/LSP1^+^, *Spi1*, *Cebpd*, *Irf5*, *Irf8*	Immune-related Functions (Phagocytosis, Expansion under stimuli, antigen presentation and T-cell activation), Not platelet-producing
HSC niche-supporting MKs	8N–32N	MYLK4^+^, *Igf1*, *Pf4*, *Wnt3a*, *Wnt4*, *Dkk1*, *Dkk2*	HSC regulation
Platelet-producing MKs	>32N	*Tubb1*, *Myh9*, *Vwf*, *Gp1ba*, *Gp5*, *Gp6*, *P2yr1*, *P2yr12*	Platelet production, hemostasis
Polyploidization MKs	2N–32N	*Pola2*, *Pold2*	Cell cycle regulation

**Table 2:** Surface receptors and ploidy status of Bone Marrow and Extramedullary (Lung/Spleen) Megakaryocytes.

Ploidy Status and Surface Markers	Bone Marrow MKs	Extramedullary MKs (Lung/Spleen)	References
Ploidy status	2–128N	2–16N (low-ploidy cells)	[7,9,52]
IL-6R	**✔**	**✔**	[5,56]
TLR	**✔**	**✔**	[55,57]
HLA-DR	**✔**	**✔**	[52]
IFNAR1/2	**✔**	**✔**	[58]
LFA-1	**✔**	**✔**	[14,52]
CD11a	**✔**	**✔**	[52]
CD80/CD86	**✔**	**✔**	[52,59]
MHC-I	**✔**	**✖**	[59,60]
ICAM-1	**✔**	**✔**	[52]
CLR	**✔**	**✔**	[60,61,62]
CD40L	**✔**	**✔**	[52,63]
GPIIb/IIIa	**✔**	**✔**	[64,65]
c-MPL	**✔**	**✔**	[66,67,68]
FcγRIIA	**✔**	**✔**	[5,69]
GPIb-IX-V	**✔**	**✔**	[5,70]
MHC-II	**✖**	**✔**	[52,71]
CCR7	*	**✔**	[52]

* = low levels. **✔** = surface marker is present; **✖** = surface marker is absent.

**Table 3:** Megakaryocyte Responses to Pathogens.

Pathogen	Mechanism	Outcomes	Classification
*Klebsiella pneumoniae*	Platelet activation → apoptosis → inhibited MK maturation	↓ Platelets, ↑ bleeding	Thrombocytopenia-inducing
*Fusobacterium nucleatum*	Aberrant MK maturation → excessive thrombosis	↑ Clotting	Thrombosis-inducing
*Pseudomonas aeruginosa*	MK cytotoxicity via p38 → reduced platelet count	↓ Platelets	Thrombocytopenia-inducing
*Bacillus anthracis*	Downregulation of *DACH1* → reduced platelet production	↓ Platelets	Thrombocytopenia-inducing
*Escherichia coli*	Internalization via MHC receptors → antigen processing	↑ Antigen Presentation	Activation

↓ = decrease, ↑ = increase.

## Data Availability

No new data were created or analyzed in this study. Data sharing is not applicable to this article.

## Non-Standard Abbreviations and Acronyms

AMR	Ashwell–Morell receptor
BM	bone marrow
CLEC-2	C-type lectin-like receptor 2
c-MPL	myeloproliferative leukemia protein
CXCL2/MIP-2	chemokine (C-X-C motif) ligand 2/macrophage inflammatory protein-2
CXCL4/PF4	chemokine (C-X-C motif) ligand 4/platelet factor 4
DAMPs	damage-associated molecular patterns
DIC	disseminated intravascular coagulation
DMS	demarcation membrane system
FGF-4	fibroblast growth factor-4
G-CSF	granulocyte colony-stimulating factor
GP	glycoprotein
HMGB-1	high mobility group box 1
HSCs	hematopoietic stem cells
HSPCs	hematopoietic stem and progenitor cells
IFITM3	interferon-induced transmembrane protein 3
iPSCs	inducible pluripotent stem cells
ITP	idiopathic thrombocytopenic purpura
LAMP-1	lysosome-associated membrane protein 1
LNP	lipid nanoparticles
LPS	lipopolysaccharide
MEPs	megakaryocyte-erythroid progenitors
MHC	major histocompatibility complex
MK	megakaryocyte
MkPs	megakaryocyte precursors
MPPs	multipotent progenitors
NLRs	NOD-like Receptors
PAMPs	pathogen-associated molecular patterns
PS	phosphatidylserine
RANTES	regulated on activation, normal T cell expressed, and secreted
ROS	reactive oxygen species
S1P	sphingosine-1-phosphate
SDF-1	stromal cell-derived factor-1
SIC	sepsis-induced coagulopathy
TGFβ	transforming growth factor beta
TLR	toll-like receptor
TNF-α	tumor necrosis factor alpha
TPO	thrombopoietin
